# Non-Homogeneous Toxicity of Polyvinyl Chloride Microplastics on Microalgal Cells Regulated by Nutrient Availability

**DOI:** 10.3390/biology15120906

**Published:** 2026-06-10

**Authors:** Chun Wang, Zhongquan Jiang, Jingyu Ma, Meilin He

**Affiliations:** 1 College of Oceanography, Hohai University, Nanjing 210024, China; 20230943@hhu.edu.cn; 2 College of Resources and Environmental Science, Nanjing Agricultural University, Nanjing 211800, China; majingyu2000@outlook.com; 3 Jiangsu Provincial Key Laboratory of Coastal Saline Soil Resources Utilization and Ecological Conservation, Nanjing Agricultural University, Nanjing 211800, China; 4 Key Laboratory of Environmental Health Impact Assessment of Emerging Contaminants, Ministry of Ecology and Environment, School of Environmental Science and Engineering, Shanghai Jiao Tong University, Shanghai 200240, China; zhongquanj@sjtu.edu.cn; 5 East China Sea Fisheries Research Institute, Chinese Academy of Fishery Sciences, Shanghai 200090, China

**Keywords:** microplastics, toxic effect, microalgae, nutrient limitation, interaction effect

## Abstract

Microplastics (MPs) have been shown to exert time-dependent toxic effects on microalgae, with toxicity either intensifying or alleviating over prolonged exposure. However, the underlying mechanisms driving this phenomenon remain unclear, particularly regarding the role of external nutrient depletion in the surrounding medium. Herein, we performed bioassays and computational modeling to evaluate the effects of polyvinyl chloride microplastics (mPVC, 10, 50, and 100 mg L^−1^) on the growth and physiology of *Desmodesmus* sp. under varying initial medium nutrient levels (100%, 25%, 8%, and 4%) for 96 h. The results demonstrated that nutrient levels in the culture medium modulated the toxic impact of mPVC. Under a 25% nutrient concentration, exposure to 100 mg L^−1^ mPVC significantly inhibited algal growth by 6.5%, whereas at a 4% nutrient level, the growth of *Desmodesmus* sp. was significantly enhanced by 7.0%. Consistently, mPVC exposure at 25% nutrient concentration led to a sharper decline in intracellular pigment content (13.1–15.5%), soluble sugars content (8.2–27.0%), and photosynthetic efficiency (4.3–20.5%). In contrast, exposure to mPVC at 4% nutrient level improved the light use efficiency (11.9–15.4%) and electron transport rate (9.2–13.9%) of the algal cells. Independent action modeling revealed that as medium nutrient levels decreased, the interaction effects of mPVC exposure shifted from a synergistic to an antagonistic relationship. Overall, our findings identify medium nutrient availability as a critical regulatory factor influencing the toxic effects of mPVC on microalgae, potentially providing different insights into understanding the time-dependent toxicity of MPs.

## Simple Summary

Microplastics pollution threatens aquatic organisms, but what is puzzling is why the toxic effects of microplastics on microalgae become stronger or weaker with prolonged exposure. A key suspect is the gradual depletion of nutrients as algae grow. We examined whether nutrient levels alter the impact of polyvinyl chloride microplastics on *Desmodesmus* sp. We cultured the algal cells with nutrient supplies ranging from rich to extremely poor (100%, 25%, 8%, and 4% of initial medium nutrient levels) and added different amounts of polyvinyl chloride microplastics. Under moderately low nutrients (25% nutrient level), the microplastics reduced growth and impaired photosynthesis. However, under severely nutrient-depleted conditions (4% nutrient level), the same microplastics enhanced growth and improved the algae’s light-use efficiency. A mathematical model for combined stress revealed that as nutrients dwindled, the interaction switched from synergistic harm to antagonism. This finding demonstrates that nutrient availability can dictate whether microplastics are harmful or beneficial, potentially offering a different perspective for understanding time-varying microplastic toxicity.

## 1. Introduction

Plastics have become integral to daily human life due to their durability, low cost, and ease of manufacturing. In 2024, global plastic production reached 430.9 million tons [1]. However, only approximately 9% of plastic waste is recycled, 12% is incinerated, and the vast majority (79%) accumulates in landfills or the natural environment [2]. Through processes such as mechanical fragmentation, photo-oxidation, thermal degradation, and biodegradation, plastic waste gradually breaks down into smaller fragments [3]. Plastic particles, fibers, or films with diameters less than 5 mm are classified as microplastics (MPs) [4]. Research estimates that 10 to 40 million tons of MPs are released into the environment annually [5]. The widespread presence of MPs across various environmental media and biological systems has prompted significant concerns regarding their potential ecological and human health impacts [5,6,7].

Microalgae are vital primary producers in aquatic ecosystems [8]. MPs introduced into water bodies can disrupt the physiological functions and biochemical responses of algal cells, adversely affecting their growth and metabolic activities and ultimately leading to toxicity [8,9]. Studies have demonstrated that polystyrene microplastics (PS-MPs) significantly inhibit the growth of *Skeletonema costatum*, with the inhibitory effects intensifying with prolonged exposure (from day 1 to day 4) [10]. Conversely, the chlorophyll fluorescence parameters of *Phaeodactylum tricornutum* are more rapidly disrupted upon initial exposure to PS-MPs but recover to baseline levels over time (from day 4 to day 7) [11]. Mao et al. observed that the growth of *Chlorella pyrenoidosa* was inhibited during early exposure to PS-MPs over an extended period (30 days), followed by a recovery and even enhanced growth [12]. A comparable pattern was also reported during the long-term (45 days) exposure of *Microcystis aeruginosa* to polylactic acid microplastics (PLA-MPs) [13]. The findings suggest that the toxic effects of MPs on microalgae are time-dependent, exhibiting either exacerbation or alleviation of toxicity with prolonged exposure. These effects have been primarily attributed to changes in cellular behaviors, such as cell wall thickening, homo-/hetero-aggregation, and enhanced photosynthesis or photosynthetic carbon assimilation [12,13]. However, an often-overlooked external factor is the depletion of nutrients in the culture medium as algal cells grow. After 7 days of *Chlorella pyrenoidosa* cultivation, the total phosphorus content in BG-11 medium decreased to just 37.69% of its initial concentration [14], demonstrating dynamic nutrient fluctuations in the microenvironment. Previous studies have shown that nutrients modulate the biological impacts of heavy metals, such as cadmium and zinc, on benthic diatom communities [15]. Meanwhile, Jiang et al. confirmed that the environmental stressors arsenic and lead produce a combined toxic effect on Chlamydomonas reinhardtii and accurately predicted the toxicity of the two pollutants at different concentrations using independent action (IA) and concentration addition (CA) model tools [16]. A critical unresolved question is whether medium nutrient levels act as a regulatory factor in the toxicity of MPs to microalgae. For instance, while microalgae may exhibit stronger resilience to MPs stress when nutrient levels are sufficient, they could become more sensitive under nutrient-limited conditions. In this study, we unexpectedly found that polyvinyl chloride microplastics promote the growth and photosynthetic metabolism of algal cells under conditions of severe nutrient limitation, challenging the conventional understanding of the harmful effects of microplastics and highlighting the dual nature and complexity of their biological effects on microalgae. Therefore, addressing this question could enhance our understanding of the mechanisms driving the time-dependent toxicity of MPs on microalgae.

Algal photosynthesis is critical for oxygen production and the biogeochemical cycles of nitrogen and phosphorus [8,12]. However, the photosynthetic systems of microalgae are highly sensitive to MP stress [17]. This sensitivity could be attributed to the water-insoluble nature of MPs particles, which can reduce visible light transmittance, decreasing the light flux reaching the algal cell surface and directly disrupting photosynthesis [14,18]. The shading effect has been identified as the primary mechanism underlying the toxic effects of MPs on microalgae, particularly for larger MP particles [9,19]. Liu et al. demonstrated that larger MPs (2 μm vs. 0.1 μm) block environmental light transmission, impairing photosynthesis and causing toxicity in algal cells [19]. Conversely, microalgae have been shown to mitigate MPs’ stress by adjusting chlorophyll fluorescence parameters and altering carbohydrate content in photosynthetic products [17]. When external nutrients, such as nitrogen and phosphorus, are depleted, the photosynthetic process is altered [20,21], further influencing the toxicity of MPs and the adaptive capacity of microalgal cells to MP stress. However, how exposure to MPs affects the photosynthetic responses of microalgae under varying medium nutrient levels remains poorly understood. Further research is necessary to elucidate the regulatory mechanisms by which nutrient availability modulates the effects of MPs on algal cells.

The microalga *Desmodesmus* sp. is widely distributed in natural aquatic ecosystems [22,23] and plays a key role in shaping species competition within phytoplankton communities [24]. Polyvinyl chloride plastics account for 19% of total global plastic production [25] and exhibit significant toxicity to microalgae [9]. Additionally, larger microplastic particles (100–200 μm) represent a substantial proportion of microplastics found in natural water bodies [26]. Consequently, this study selected polyvinyl chloride microplastics (mPVC, approximately 150 μm in diameter) and *Desmodesmus* sp. as the test objects to investigate the effects of mPVC on algal growth, pigment content, chlorophyll fluorescence parameters, and soluble sugar content under varying initial medium nutrient levels (100%, 25%, 8%, and 4%). Furthermore, model calculations were performed to evaluate the interactive toxicity between mPVC and varying nutrient levels. The primary objectives of this study were to: (1) elucidate the responses and differences in microalgal cells exposed to MPs under varying initial nutrient conditions, and (2) uncover the processes and mechanisms by which nutrient levels regulate the toxic effects of MPs on microalgae. The results contribute to a broader understanding of the time-dependent toxicity of MPs on microalgae and provide a theoretical foundation for the ecological risk assessment of MPs.

## 2. Materials and Methods

### 2.1. Microalgal Strain and Reagents

**Figure 1:**
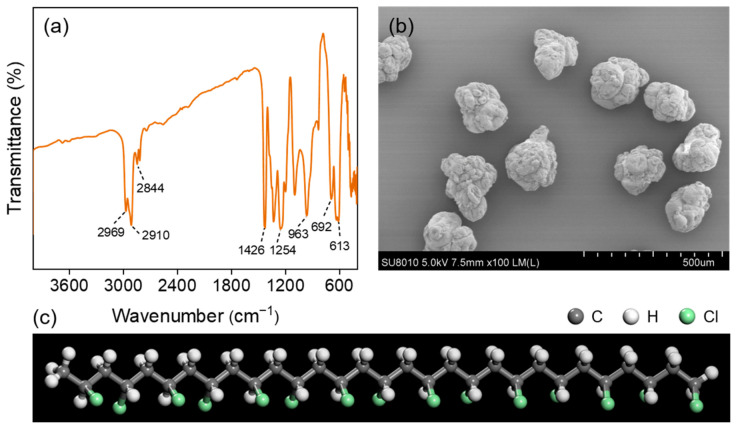
Infrared spectrum (**a**), scanning electron microscopy image (**b**), and chemical structure formula ((**c**), 15 repeating units) of mPVC.

*Desmodesmus* sp. was obtained and preserved at the Jiangsu Key Laboratory of Marine Biology, Nanjing Agricultural University. The microalga was precultured in sterile BG-11 medium in an incubator at 25 ± 1 °C under a light intensity of 45 μmol·m^−2^·s^−1^ supplied by cool-white fluorescent tubes with the light-dark cycle of 14 h: 10 h. Microparticles of mPVC (Figure 1), with a density of 1.4 g cm^−3^, were purchased from Shanghai Aladdin Bio-Chem Technology Co., Ltd. (Shanghai, China). A working mPVC suspension (5 g L^−1^) was prepared in sterile deionized water after pre-disinfecting the particles with 75% (*v*/*v*) ethanol and repeatedly rinsing them with sterile deionized water. To ensure proper dispersion, the solution was ultrasonicated for 10 min before commencing the experiment.

### 2.2. Experiment Design

**Figure 2:**
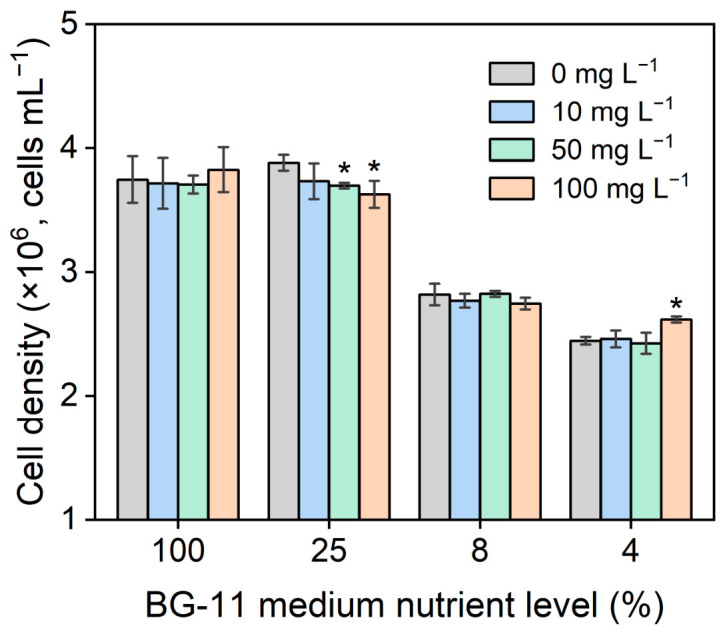
Changes in the cell growth of *Desmodesmus* sp. with and without mPVC under different medium nutrient levels. Asterisks indicate significant differences between exposure groups and the control group (* *p* < 0.05; the specific values, listed from left to right, are 0.038, 0.021, and 0.017).

**Figure 3:**
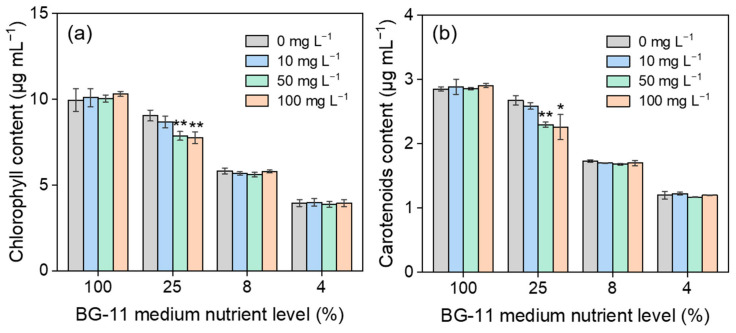
Changes in the chlorophyll (**a**) and carotenoids (**b**) contents of *Desmodesmus* sp. with and without mPVC under different medium nutrient levels. Asterisks indicate significant differences between exposure groups and the control group (* *p* < 0.05, ** *p* < 0.01).

To investigate the differences in the toxicity of MPs on microalgae under varying medium nutrient levels and to determine whether nutrient limitation exacerbates or mitigates the impact of MPs on microalgal growth and physiology, BG-11 medium was used as a baseline at 100% level (Table 1). Four initial nutrient gradients were established through the proportional dilution of all components: 100%, 25%, 8%, and 4%, representing conditions of normal nutrition, mild nutrient limitation, moderate nutrient limitation, and severe nutrient limitation, respectively (Figure 2 and Figure 3). During the experiment, algal cells in the logarithmic growth phase were harvested by centrifugation at 3000 rpm for 5 min. The cells were resuspended in sterile medium corresponding to each gradient and washed three times. Subsequently, the washed algal cells were inoculated into the nutrient-gradient-specific medium and pre-cultured for 48 h under the same conditions used for strain activation (Section 2.1). The acclimated algal suspension was then transferred into fresh medium with the same nutrient gradients. The initial cell density was standardized to 2.0 × 10^5^ cells⋅mL^−1^, and the pH was adjusted to 7.4. mPVC standard working solutions were added to achieve final concentrations of 0 (control group), 10, 50, and 100 mg L^−1^. These concentrations were selected based on their environmental relevance and to encompass relatively high exposure levels [12,17]. Furthermore, establishing exposure levels that extend from low to high doses producing toxicity enhances our capacity to examine the regulatory role of nutrient availability in the biological effects of MPs. This approach mitigates the risk of overlooking objectively observable phenomena and patterns that may emerge when exposure concentrations are below the maximum non-lethal dose. Each treatment was conducted in triplicate, and bioassays were performed after 96 h of exposure. Throughout the experiment, all treatment groups were manually shaken at least three times daily to promote contact between algal cells and mPVC, while minimizing shear forces and physical collisions associated with continuous agitation, such as that produced by an orbital shaker. This approach was necessary to prevent potential alterations in the surface physiological characteristics of the algal cells. Additionally, their positions were randomly rotated to maintain uniform culture conditions.

### 2.3. Characterization of mPVC Particles

An appropriate amount of mPVC particles was placed into a clean glass Petri dish and dried at 40 °C for 4 h to remove surface moisture. The functional groups of mPVC were analyzed in transmission mode using a Micro-Fourier-transform infrared spectrometer (μ-FTIR, Thermo Fisher Scientific Nicolet iN10, Waltham, MA, USA) over the wavenumber range of 4000–400 cm^−1^, with a resolution of 4 cm^−1^ and 64 scans. The morphology and particle size of mPVC were examined using scanning electron microscopy (SEM, Hitachi SU8010, Tokyo, Japan). Before imaging, the mPVC particles were coated with a gold film via ion sputtering and observed at an acceleration voltage of 5.0 kV.

### 2.4. Measurement of Cell Density and Pigment Content

The optical density (OD) of the algal cells was measured at a wavelength of 680 nm using a UV-Vis spectrophotometer (Model P1, MAPADA Instruments, Shanghai, China). To minimize the potential interference of mPVC particles in absorbance measurements, a 10 mL sample of the algal suspension was transferred to a 15 mL centrifuge tube and allowed to settle for 5 min. The algal cells could maintain a uniformly suspended state during this period, with a decrease in absorbance of less than 1%. Subsequently, a pipette was used to carefully extract the upper middle layers of the algal solution for absorbance measurement. The cell density was calculated using a standard calibration curve relating cell count to OD_680_: cell density = (6.321 × OD_680_ − 0.180) × 10^6^ (R^2^ = 0.9972).

Pigments from *Desmodesmus* sp. were extracted using the methanol method. A 4 mL sample of the algal suspension was centrifuged at 8000 rpm for 10 min at 4 °C. An equal volume of methanol was added to the pellet, followed by vigorous shaking, and the mixture was left overnight at 4 °C in a refrigerator. The mixture was then centrifuged again at 8000 rpm for 10 min at 4 °C. Absorbance values (A) of the supernatant were measured at wavelengths of 666 nm, 653 nm, and 470 nm. Pigment concentrations (expressed in μg mL^−1^) were calculated using the following formula:

Chl a = 15.65 × A_666_ − 7.34 × A_653_ (1)

Chl b = 27.05 × A_653_ − 11.21 × A_666_ (2)

Car = (1000 × A_470_ − 2.86 × Chl a − 12.92 × Chl b)/245 (3)

where Chl a, Chl b, and Car indicate the contents of chlorophyll a, chlorophyll b, and carotenoids, respectively, while A_666_, A_653_, and A_470_ represent the absorbance values of the sample at 666 nm, 653 nm, and 470 nm, respectively.

### 2.5. Determination of Chlorophyll Fluorescence Parameters

The chlorophyll fluorescence parameters of *Desmodesmus* sp. were measured using a pulse-amplitude-modulated fluorometer (Phyto-PAM Walz, Effeltrich, Germany). The algal suspension was appropriately diluted to ensure the chlorophyll a concentration did not exceed 300 μg mL^−1^ and was dark-adapted for 15 min. Subsequently, 2 mL of the suspension was placed in a quartz cuvette and positioned in the PHYTO-ED detector. Once the light signal stabilized, the maximum (F_v_/F_m_) and effective (Φ_PSII_) photochemical quantum yield of PSII reaction centrals were measured. The photosynthetically active radiation (PAR) was varied between 32 and 2064 μmol·m^−2^·s^−1^, and rapid light-response curves (RLCs) were generated, with each PAR level applied for 20 s. The initial slope of the RLC, representing light use efficiency (α), was calculated using the Platt fitting equation. The semi-saturation light intensity (I_k_) was determined using the formula:

I_k_ = rETR_max_/α. (4)

### 2.6. Analysis of Soluble Sugars Content

A 5 mL sample of algal suspension was centrifuged at 8000 rpm at 4 °C for 10 min. The pellet was resuspended in an equal volume of deionized water. The algal cells were then disrupted using an ultrasonic cell disruptor (Scientz-IID, Ningbo Scientz Biotechnology Co., Ltd., Shanghai, China; power: 540 W; on time: 5 s, off time: 10 s; total duration: 10 min). Following disruption, the sample was centrifuged again at 8000 rpm for 10 min at 4 °C. One milliliter of the supernatant was accurately pipetted into a 10 mL transparent centrifuge tube, to which 0.5 mL of 6% (*w*/*v*) phenol solution and 2.5 mL of concentrated sulfuric acid were added. The mixture was thoroughly mixed and incubated in a water bath at 45 °C for 30 min. After cooling to room temperature, the content of soluble sugars was measured at 490 nm.

### 2.7. Calculation of Toxic Action Modes

The toxicity interaction of mPVC and different medium nutrient levels was identified using the following equation [27]:

(5) $$E({Cx}_{mix})=1-\prod_{i=1}^{n}(1-E\left({\mathrm{Cx}}_{i}\right))$$

where E(Cx_mix_) represents the total growth inhibition rate (%) of n substances, while E(Cx_i_) denotes the growth inhibition rate (%) of the i-th substance at a concentration of C_i_. The type of combined toxicity is inferred by comparing the expected results calculated using the IA model (EC_expected_) with the experimental results (EC_tested_) [27]. If EC_tested_ exceeds EC_expected_, the toxic interaction is classified as synergistic. Conversely, if EC_tested_ is lower than EC_expected_, the interaction is considered antagonistic. When EC_tested_ equals EC_expected_, the interaction is defined as additive.

### 2.8. Data Processing and Statistical Analysis

The inhibition ratio of *Desmodesmus* sp. by mPVC was calculated by the following equation:

Inhibition ratio (%) = (1 − T_i_/C_i_) × 100% (6)

where C_i_ and T_i_ represent the values of the i-th bioassay parameter in the control group and the treatment group, respectively.

The means and standard deviations were calculated from three replicates for each treatment group. One-way analysis of variance (ANOVA) followed by Tukey’s post hoc multiple comparisons test was conducted using GraphPad Prism software (version 8.0.2). Statistical significance was determined at a *p*-value of less than 0.05.

## 3. Results

### 3.1. Physicochemical Properties of mPVC

The mPVC was analyzed and characterized using Fourier transform infrared spectroscopy (FTIR) and scanning electron microscopy (SEM). As shown in Figure 1a, the FTIR spectrum of mPVC displays distinct characteristic peaks at 692 cm^−1^ and 613 cm^−1^, corresponding to the strong absorption of C-Cl bonds [28]. Additionally, several characteristic peaks associated with C-H and C-C bonds were identified at 2969 cm^−1^, 2910 cm^−1^, 2844 cm^−1^, 1426 cm^−1^, 1254 cm^−1^, and 963 cm^−1^ [28,29]. SEM analysis reveals that mPVC particles possess an irregular morphology with a rough surface texture and an approximate particle size of 150 μm (Figure 1b). Furthermore, Figure 1c illustrates the chemical structure of PVC plastic, primarily consisting of C, H, and Cl elements, which are covalently bonded to form C-C, C-H, and C-Cl bonds. These findings are consistent with the FTIR results shown in Figure 1a, confirming the accurate identification of the plastic material.

### 3.2. Effect of mPVC on the Growth of Desmodesmus sp. Under Different Medium Nutrient Levels

The impact of mPVC on the growth of *Desmodesmus* sp. at varying nutrient levels in BG-11 medium is shown in Figure 2. As medium nutrient levels decreased, the cell density of *Desmodesmus* sp. initially remained stable but then gradually declined. Compared to the control group, the presence of mPVC led to a 3.8–6.5% reduction in cell density at the 25% nutrient level. Interestingly, when the medium nutrient level was reduced to 4%, exposure to 100 mg L^−1^ mPVC significantly enhanced algal growth, resulting in a 7.0% increase in cell density. At nutrient concentrations of 100% and 8%, mPVC exhibited no statistically significant influence on the growth of *Desmodesmus* sp. within the tested concentration range.

### 3.3. Effect of mPVC on the Chlorophyll and Carotenoids Contents of Desmodesmus sp. Under Different Medium Nutrient Levels

Consistent with the trend in cell density (Figure 2), the contents of chlorophyll (Figure 3a) and carotenoids (Figure 3b) in *Desmodesmus* sp. cells remained stable as medium nutrient concentrations decreased from 100% to 25%, but declined as nutrient levels dropped further to 4%. The addition of mPVC had minimal influence on chlorophyll and carotenoid contents at nutrient levels of 100%, 8%, and 4%. However, at the 25% nutrient concentration, higher mPVC doses (50 and 100 mg L^−1^) significantly reduced chlorophyll and carotenoid contents in the algal cells compared to the control group, with reductions of 13.1–14.2% and 14.1–15.5%, respectively.

### 3.4. Effect of mPVC on the Chlorophyll Fluorescence Parameters of Desmodesmus sp. Under Different Medium Nutrient Levels

**Figure 4:**
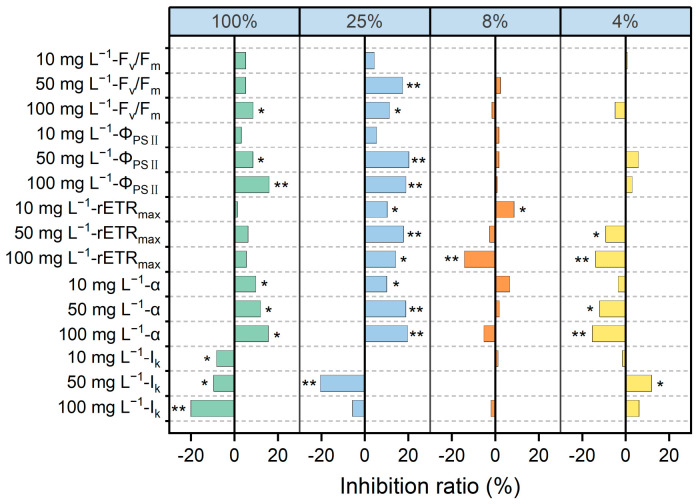
Inhibition ratio of chlorophyll fluorescence parameters in *Desmodesmus* sp. exposed to varying concentrations of mPVC under different medium nutrient levels. Asterisks indicate significant differences between exposure groups and the control group (* *p* < 0.05, ** *p* < 0.01). Percentages of 100%, 25%, 8%, and 4% denote the medium nutrient levels. The numbers 10, 50, and 100 represent the concentrations of mPVC (mg L^−1^).

Figure 4 shows the changes in chlorophyll fluorescence parameters of *Desmodesmus* sp. exposed to mPVC under varying medium nutrient levels. Key parameters analyzed include the maximum photochemical quantum yield of PSII reaction centrals (F_v_/F_m_), effective photochemical quantum yield of PSII reaction centrals (Φ_PSII_), maximum relative electron transport rate (rETR_max_), light use efficiency (α), and half-saturation light intensity (I_k_). At a 100% nutrient level, mPVC exposure reduced F_v_/F_m_, Φ_PSII_, and α values by 3.2% to 16.0%, while increasing I_k_ by 8.2% to 20.1%. These effects were more pronounced at higher mPVC concentrations. At 25% nutrient level, in addition to reductions in F_v_/F_m_, Φ_PSII_, and α, rETR_max_ was also inhibited, with suppression rates ranging from 4.3% to 20.3%. Moreover, I_k_ increased significantly by 20.5% at an mPVC concentration of 50 mg L^−1^. In contrast, at an 8% nutrient level, only rETR_max_ exhibited significant variation, with decreases of 8.6% and increases of 14.2% observed at 10 mg L^−1^ and 100 mg L^−1^ mPVC concentrations, respectively. High mPVC concentrations (50 and 100 mg L^−1^) led to significant enhancements in rETR_max_ (9.2–13.9%) and α (11.9–15.4%) when the medium nutrient level was reduced to 4%, while 50 mg L^−1^ of mPVC substantially inhibited I_k_ by 11.9%.

### 3.5. Effect of mPVC on the Soluble Sugars Content of Desmodesmus sp. Under Different Medium Nutrient Levels

**Figure 5:**
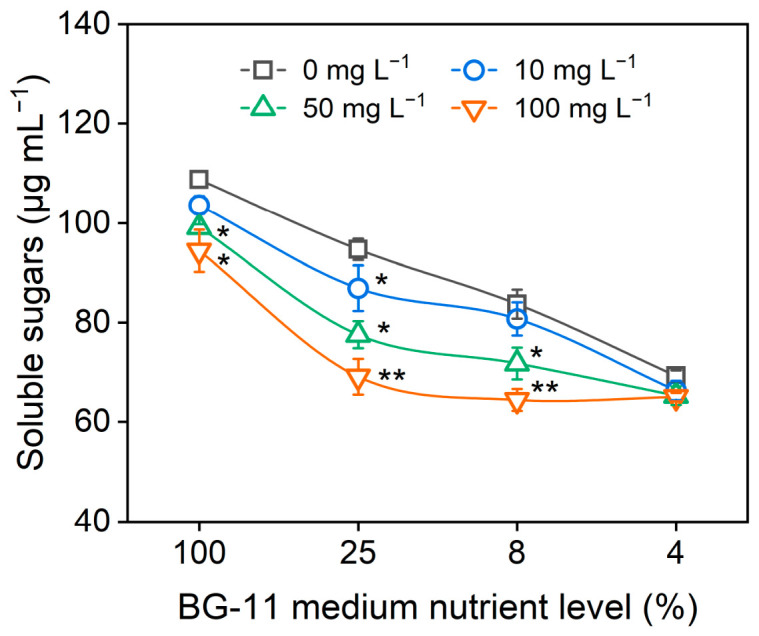
Changes in the soluble sugar content of *Desmodesmus* sp. with and without mPVC under different medium nutrient levels. Asterisks indicate significant differences between exposure groups and the control group (* *p* < 0.05, ** *p* < 0.01).

As shown in Figure 5, the intracellular soluble sugar content of *Desmodesmus* sp. decreases progressively with the reduction in nutrient levels in the medium. Under nutrient conditions of 100%, 25%, and 8%, exposure to high concentrations of mPVC (50 and 100 mg L^−1^) resulted in significant reductions in soluble sugar content of 8.8–13.2%, 18.1–27.0%, and 14.3–23.1%, respectively. Notably, even at a lower mPVC concentration of 10 mg L^−1^, a significant reduction of 8.2% in soluble sugar content was observed at the 25% nutrient level. Conversely, at the lowest nutrient level of 4%, mPVC exposure did not significantly affect the soluble sugar content of *Desmodesmus* sp. across the tested concentration range.

### 3.6. Toxicity Interaction of mPVC and Different Medium Nutrient Levels on Desmodesmus sp.

**Figure 6:**
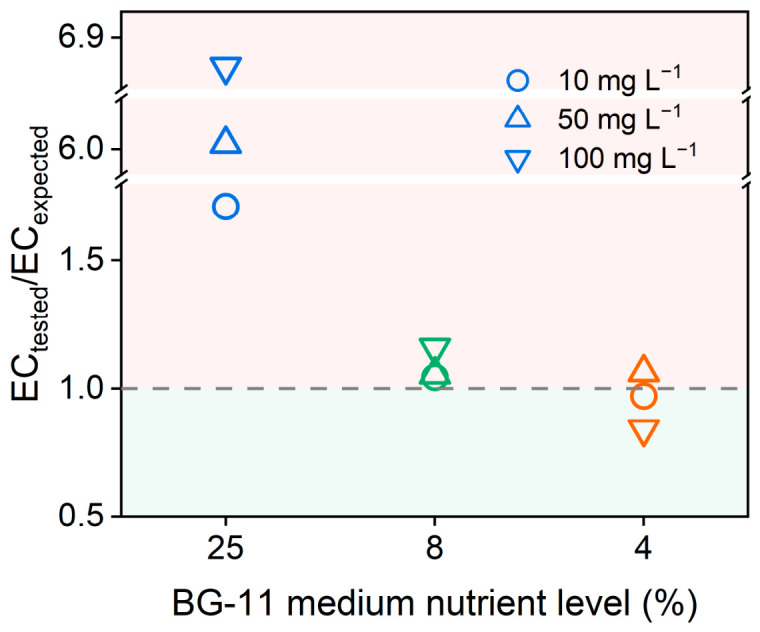
The ratios of the bioassay results to the predicted results based on the IA model. The blue, green, and orange symbols represent mPVC exposures of 10, 50, and 100 mg L^−1^, respectively.

The toxicity interaction between mPVC and varying medium nutrient levels was evaluated using the IA model, with the 100% nutrient level serving as the reference. Table 2 presents the EC_tested_, EC_expected_, and their ratio, while Figure 6 illustrates the distribution trend of E EC_tested_/EC_expected_ values. Using the assessment criteria outlined in Section 2.7, the interactive effects on the growth of *Desmodesmus* sp. were classified as strong synergy at the 25% nutrient level, weak synergy at the 8% nutrient level, and either antagonism (50 and 100 mg L^−1^ mPVC) or weak synergy (10 mg L^−1^ mPVC) at the 4% nutrient level. Overall, as nutrient levels in the medium progressively decreased, the interaction effects of mPVC exposure transitioned from synergy to antagonism.

## 4. Discussion

Numerous studies have demonstrated that MPs can disrupt the physiological functions and biochemical responses of microalgal cells through mechanisms such as the shading effect, ultimately impacting microalgal growth and metabolic activity while exerting time-dependent toxicity effects [10,11,12,13]. However, the underlying causes of this time-dependent toxicity are predominantly attributed to variations in algal cell behavior [12,13]. As cells proliferate over time, nutrients in the culture medium are rapidly depleted [14], leading to significant reductions in nutrient availability. This highlights that the nutrient content within the microenvironment where MPs interact with algal cells undergoes dynamic fluctuations. Nonetheless, it remains unclear whether external changes in nutrient levels influence the biological effects of MPs on algal cells and, consequently, serve as potential regulatory factors in the time-dependent toxicity of MPs on microalgae. Clarifying this relationship warrants further investigation. This study simulated different stages of nutrient dynamic decay over time by co-culturing *Desmodesmus* sp. with polyvinyl chloride microplastics under varying initial nutrient levels (100%, 25%, 8%, and 4%). This approach allowed us to analyze the impact of nutrient availability on the biological effects of microplastics, thereby offering a potential mechanistic explanation for their time-dependent toxicity.

Our study found that the growth of *Desmodesmus* sp. was significantly inhibited under mPVC exposure (50 and 100 mg L^−1^) when the nutrient levels in the medium decreased from 100% to 25% (Figure 2), suggesting that this transition exacerbates the toxicity of mPVC. Notably, when the medium nutrient level was further reduced to 4%, the presence of mPVC (100 mg L^−1^) instead promoted algal cell growth (Figure 2), indicating that the toxicity of mPVC was alleviated or even disappeared under extreme nutrient limitations. This dual effect of inhibition and promotion underscores the pivotal role of nutrient availability in regulating the interactions between MPs and microalgal cells and highlights its regulatory influence on the toxicity of mPVC to microalgae. Additionally, the IA model [27] theoretically demonstrated that as nutrient levels progressively decrease, the interactive effects of nutrient limitation and mPVC exposure shift from synergistic to antagonistic (Figure 6 and Table 2), aligning with the findings of the bioassay (Figure 2). Similar transitions in toxicity have been reported for environmental stressors, such as the combined toxic effects of arsenic and lead [16] or MPs and dibutyl phthalate [30], on microalgae. These observations collectively emphasize that the toxic effects of pollutants, including MPs, on microalgal cells are not static but instead undergo dynamic changes depending on the environmental conditions of the interaction.

Algal photosynthesis is highly sensitive to MPs exposure [17] and represents a key target for the toxic effects of MPs on microalgal cells [14,18]. Simultaneously, the regulation of photosynthesis serves as a crucial metabolic pathway through which microalgae resist MP-induced stress [17]. In this study, we investigated how photosynthetic responses in microalgal cells exposed to MPs vary under different medium nutrient levels, aiming to uncover potential mechanisms of toxic regulation. When compared to a medium nutrient level of 100%, exposure to mPVC under a nutrient level of 25% caused a significant reduction in intracellular chlorophyll content in *Desmodesmus* sp. (Figure 3). Moreover, it more severely inhibited photosynthetic parameters such as F_v_/F_m_, Φ_PSII_, rETR_max_, and α values (Figure 4), indicating disruption of photosynthetic processes [14,17] and subsequent growth limitations (Figure 2). The disturbance of photosynthesis parameters also indicates that during this period, the main toxic mechanism of mPVC is the shading effect. However, at a further reduced nutrient level of 4%, high concentrations of mPVC (50 and 100 mg L^−1^) activated stress defense mechanisms in *Desmodesmus* sp., enhancing light use efficiency (α) and maximum relative electron transport rate (rETR_max_) (Figure 4), which supported improved photosynthesis [31,32]. Additionally, the reduction in half-saturation light intensity (I_k_) lowered the algal cells’ demand for light flux, potentially increasing light absorption capacity [33] under the combined stress of 4% nutrient levels and mPVC exposure. Due to extreme nutrient deficiency, algal cells may allocate precious resources to maintain essential growth and metabolic processes, making it difficult for them to engage in more resource-intensive activities, such as transporting synthesized polysaccharides and proteins to the extracellular environment to form extracellular polymeric substances with adhesive properties. Therefore, at 4% nutrient level, the cell surface physiology of *Desmodesmus* sp. may also change, reducing direct contact with microplastic particles and ultimately decreasing the associated toxicity. These ultimately alleviated mPVC toxicity and promoted the growth of *Desmodesmus* sp. (Figure 2). It is noteworthy that, unlike the direct impact of shading effects induced by mPVC at a 25% nutrient level, the shading effect at this stage functions primarily as an indirect factor, resulting in effects akin to hormesis.

Polysaccharides, as key products of photosynthesis, perform various vital roles in microalgal cells [34]. On one hand, microalgal polysaccharides are highly sensitive to environmental stress, with changes in their content serving as indicators of increased external stress levels [17]. On the other hand, algal cells metabolize intracellular soluble sugars to withstand stress induced by MPs, thereby maintaining normal physiological conditions [17]. At a medium nutrient level of 100%, although the photosynthetic processes of *Desmodesmus* sp. were disrupted (Figure 4), the algal cells adjusted intracellular soluble sugar levels, channeling more energy toward growth activities rather than storage [34], as evidenced by decreased sugar content (Figure 5). This might allow the growth of *Desmodesmus* sp. to remain unaffected (Figure 2). When nutrient levels decreased to 25%, mPVC caused greater disruption of photosynthetic processes in *Desmodesmus* sp. (Figure 4), possibly surpassing the self-regulatory capacity of the cells and leading to inhibited growth (Figure 2). Conversely, at a medium nutrient level of 4%, where mPVC toxicity was alleviated, the algal cells experienced a relatively low stress state and exhibited no significant changes in soluble sugar [34] levels (Figure 5). These results, from a physiological perspective, corroborate findings on cell growth (Figure 2), the regulatory role of nutrient levels in microalgal responses to mPVC exposure as calculated by the IA model (Figure 6 and Table 2), and the toxic effects of mPVC on microalgae. Furthermore, this provides additional insights into the toxic pathways of mPVC.

Overall, our study identified significant variations in the responses of microalgal cells exposed to mPVC under different medium nutrient levels. Thus, it is crucial to account for changes in external nutrient levels when evaluating the biological effects of MPs on microalgae. Ignoring this factor would undermine the accuracy of assessments regarding the magnitude and type of MP-induced toxicity, ultimately complicating efforts to precisely evaluate the interactions and effects between MPs and microalgal cells. Additionally, current research focuses on elucidating the toxic effects of MPs on algal cells and examining the role of nutrient levels in the culture medium from the perspectives of algal growth and physiology. Future studies could extend this inquiry by investigating the role of nutrients in modulating the molecular-level effects of MPs on microalgae, such as changes in enzyme activity and the expression of photosynthetic genes. Long-term exposure experiments, conducted under normal medium nutrient conditions with periodic nutrient supplementation, should also be considered to evaluate the trends in the biological effects of MPs on microalgae, thereby offering more comprehensive evidence. Moreover, this study aims to investigate the underlying regulatory mechanisms governing the time-dependent toxic effects of microplastics on microalgae under controlled conditions. To achieve this objective, indoor cultivation experiments with strict variable control are primarily utilized. Consequently, the interpretation of the biological effects of microplastics MPs in real aquatic nutrient environments is subject to inherent limitations.

## 5. Conclusions

Using algal growth assessments, physiological measurements, and model calculations, our study demonstrated the regulatory influence of medium nutrient levels on the toxic effects of MPs on microalgae. At an initial nutrient level of 25%, higher concentrations of mPVC exhibited elevated toxicity by disrupting the photosynthetic processes of *Desmodesmus* sp., significantly inhibiting its growth compared to a medium nutrient level of 100%. Conversely, at a nutrient level of 4%, mPVC exposure enhanced light use efficiency and electron transport rates in *Desmodesmus* sp., promoting cell growth. The IA model calculations revealed that as medium nutrient levels progressively decrease, the interaction effects of mPVC exposure initially display synergistic toxicity but later transition to antagonistic effects. Overall, these findings potentially provide a different perspective for understanding the time-dependent toxicity of MPs in microalgae and offer valuable theoretical references for ecological risk assessments of MPs. Nevertheless, static toxicity assessments may overestimate or underestimate the risks of microplastic pollutants, depending on the nutrient status of the receiving water body.

## Figures and Tables

**Table 1:** The components of BG-11 medium.

Component	Molecular Weight (g·mol^−1^)	Concentration (mg·L^−1^)
Sodium nitrate (NaNO_3_)	84.99	1500.0
Potassium phosphate dibasic (K_2_HPO_4_)	174.20	40.0
Magnesium sulfate (MgSO_4_·7H_2_O)	246.47	75.0
Citric acid	192.12	6.6
Ammonium ferric citrate	261.98	6.0
Ethylenediaminetetraacetic acid disodium salt (EDTA-2Na)	336.21	1.0
Sodium carbonate (Na_2_CO_3_)	105.99	20.0
Calcium chloride (CaCl_2_·2H_2_O)	147.00	36.0
Boric acid (H_3_BO_3_)	61.83	2.86
Manganese chloride (MnCl_2_·4H_2_O)	197.90	1.81
Zinc sulfate (ZnSO_4_·7H_2_O)	287.55	0.22
Sodium molybdate (Na_2_MoO_4_·2H_2_O)	205.92	0.39
Cupric sulfate (CuSO_4_·5H_2_O)	249.69	0.08
Cobalt nitrate (Co (NO_3_)_2_·6H_2_O)	291.04	0.05

**Table 2:** The bioassay results, predicted outcomes from the IA model, their ratios, and the corresponding modes of toxic action.

Nutrient Level (%)	mPVC Conc. (mg L^−1^)	EC_tested_ (%)	EC_expected_ (%)	EC_tested_/EC_expected_	Interaction Effect
25	10	7.24	4.23	1.71	Synergism
	50	8.18	1.36	6.02	Synergism
	100	9.96	1.47	6.79	Synergism
8	10	29.13	27.92	1.04	Synergism
	50	27.14	25.76	1.05	Synergism
	100	29.92	25.84	1.16	Synergism
4	10	36.47	37.58	0.97	Antagonism
	50	37.94	35.71	1.06	Synergism
	100	30.08	35.78	0.84	Antagonism

## Data Availability

Data is contained within the article.
